# Comparative impact of the affordable care act on breast cancer outcomes among women in two US states

**DOI:** 10.3389/fonc.2024.1460714

**Published:** 2024-11-07

**Authors:** Oluwasegun Akinyemi, Mojisola Fasokun, Terhas Weldeslase, Eunice Odusanya, Irene Akinyemi, Kailyn Geter, Meghana Akula, Miriam Michael, Kakra Hughes, Robin Williams

**Affiliations:** ^1^ College of Medicine, Howard University, Washington, DC, United States; ^2^ Department of Epidemiology, University of Alabama at Birmingham, Birmingham, AL, United States; ^3^ School of Nursing, Spoon River College, Canton, IL, United States

**Keywords:** Georgia, Louisiana, affordable care act, Medicaid expansion, overall mortality, cancer-specific mortality, inverse probability of treatment weighting, difference - in – differences

## Abstract

**Introduction:**

Since the implementation of the Patient Protection and Affordable Care Act (ACA) and Medicaid expansion, states that adopted the policy have seen reduced uninsured rates. However, it is unclear whether increased healthcare access, particularly for minority and socioeconomically disadvantaged groups, has translated into measurable improvements in health outcomes.

**Objective:**

Our study aims to evaluate the impact of the ACA and Medicaid expansion on breast cancer outcomes in Louisiana, which has implemented the policy, compared to Georgia, which has not, as of 2024.

**Methodology:**

We conducted a retrospective study using SEER registry data from January 2011 to December 2021, including women aged 18-64 diagnosed with breast cancer. The impact of the ACA and Medicaid expansion on cancer-specific survival (CSS), overall survival (OS), and stage at presentation was evaluated. The cohort was divided into pre-ACA (2011-2015) and post-ACA (2017-2021) periods, with a one-year washout (2016). A difference-in-difference (DID) approach compared outcomes between Louisiana and Georgia.

**Results:**

The study analyzed 62,381 women with breast cancer, with 32,220 cases in the pre-ACA period (51.7%) and 30,161 in the post-ACA period (48.3%). In Georgia, 43,279 women were included (52.3% pre-ACA vs. 47.7% post-ACA), while Louisiana had 19,102 women (50.1% pre-ACA vs. 49.9% post-ACA). Medicaid expansion in Louisiana was associated with a 0.26 percentage point reduction in overall deaths (95% CI: -10.9 to 10.4) and a 5.97 percentage point reduction in cancer-specific mortality (95% CI: -26.1 to 14.2). There was also no significant difference in disease stage at presentation compared to Georgia.

**Conclusion:**

This study found no significant differences in overall mortality, cancer-specific mortality, or disease stage at presentation among women with breast cancer in Louisiana, which implemented Medicaid expansion in 2016, compared to Georgia, which has not expanded Medicaid.

## Introduction

Breast cancer remains a significant public health concern in the United States, being one of the most commonly diagnosed cancers among women and a leading cause of cancer-related mortality (1, 2). Early detection and timely, effective treatment are critical factors influencing survival rates and overall patient outcomes (3). In recent years, healthcare policies aimed at increasing access to preventive care and medical services have become central to efforts to improve cancer outcomes (4, 5). The Patient Protection and Affordable Care Act (ACA), enacted in 2010, represents one of the most substantial healthcare reforms in U.S. history, with provisions intended to expand healthcare coverage, improve access to preventive services, and reduce healthcare disparities (6–8).

The ACA introduced several key measures, including the expansion of Medicaid, the establishment of health insurance marketplaces, and the mandate for coverage of preventive services such as mammograms without cost-sharing (9). These provisions aimed to reduce financial barriers to healthcare and encourage early detection of diseases, including breast cancer. Prior studies have suggested that these reforms could lead to improved cancer outcomes by facilitating earlier diagnosis and timely treatment (10–12). The significance of the ACA’s impact on breast cancer outcomes (CSS, OS and disease stage at presentation) cannot be overstated.

Georgia and New Jersey provide a unique opportunity to investigate the effects of the ACA. Louisiana implemented Medicaid expansion under the ACA, while Georgia did not. This study leverages this natural experiment to evaluate the ACA’s impact on breast cancer-specific survival and the stage at presentation. The objectives of this study are twofold: first, to determine whether the implementation of the ACA has led to improved breast outcomes in Louisiana compared to Georgia; and second, to assess whether there has been a shift in the stage at presentation of breast cancer diagnoses in these states. By examining these outcomes, this study aims to provide valuable insights into the effectiveness of healthcare policy reforms in improving cancer care and addressing healthcare disparities.

## Methodology

### Study design

This retrospective comparative cohort study utilized population-based cancer registry data from the SEER registry. The registry provides comprehensive data on cancer incidence, treatment, and outcomes, covering approximately 36.7% of the U.S. population (13–15).

### Study population

The study population comprised women aged 18 to 64 years diagnosed with breast cancer between January 1, 2011, and December 31, 2021, in two distinct regions: Georgia, representing a non-Expansion State, and Louisiana, representing a Medicaid Expansion State. The selection of these states facilitated a comparative analysis of the impact of Medicaid expansion on breast cancer outcomes.

### Study period and periods of interest

The study analyzed two time periods: pre-ACA (2011–2015) and post-ACA (2017–2021), with a washout period from January to December 2016 to allow for full implementation of the Medicaid expansion in Louisiana, which adopted the policy in 2016.

### Georgia vs. Louisiana

We chose Georgia and Louisiana for comparison due to their differing ACA implementation statuses, with Louisiana implementing the ACA with its Medicaid expansion component and Georgia not (8, 16, 17). Despite economic differences, both states share similar key indices crucial for cancer screening, such as cancer rates, and breast cancer screening rates, despite significant differences in uninsured rates (18, 19). The American College of Radiology recommends a breast cancer risk assessment by 25 years for all women and annual mammography screening for women at average risk starting at 40 years (20, 21).

Louisiana implemented the ACA in 2016, under which Medicaid, referred to locally as Healthy Louisiana, was expanded (22, 23). This expansion allows adults aged 19-64 years with incomes up to 138% of the federal poverty level to qualify for Medicaid (23, 24). From 2015 to 2020, Medicaid expansion recorded 468,414 new enrollments, resulting in the uninsured rate dropping from 22.7% to 8.9% (24). Louisiana does not impose work requirements as a condition for Medicaid eligibility (16).

In contrast, Georgia did not adopt the ACA but instead secured a Medicaid waiver to offer health coverage to a subset of low-income adults (25). The Georgia Pathways to coverage program provides government health insurance to individuals earning up to the federal poverty level $15,060 per year for an individual adult conditional upon verification of employment, schooling, or other qualifying activities (26).

### Primary outcome of interest

The primary outcomes of interest were cancer-specific survival (CSS), overall deaths (OS), and disease stage at presentation.

#### Cancer specific survival

The CSS served as a crucial indicator of long-term prognosis and treatment effectiveness among breast cancer patients (27). The CSS was calculated using Cox regression analysis and is defined as the mortality hazard used interchangeably for survival probability as the proportion of patients who survived per unit time (years of follow-up) after their breast cancer diagnosis. The Cox regression, also known as the proportional hazards model, is a statistical technique used to examine the association between the time until an event occurs (cancer-specific deaths) and various predictor variables (states stratified by expansion status (Georgia vs. Louisiana) and period (pre-ACA vs. post-ACA)).

#### Overall survival

Overall Survival (OS) served as a key measure of general prognosis among breast cancer patients. OS was calculated using Cox regression analysis and is defined as the mortality hazard, representing the probability of survival from any cause of death per unit of time (years of follow-up) after breast cancer diagnosis (28, 29). The Cox regression model, also known as the proportional hazards model, was used to assess the relationship between time until death (from any cause) and predictor variables, including state (stratified by expansion status: Georgia vs. Louisiana) and period (pre-ACA vs. post-ACA).

#### Disease stage at presentation

The disease stage at the time of breast cancer diagnosis was categorized into three groups based on the SEER registry’s combined summary stage (2004+). This variable allows for consistent stage analysis over time and was derived from the SEER Combined Summary Stage 2000 (2004–2017) and Derived Summary Stage 2018+ variables (30). The stage reflects how far the cancer has spread from its origin:

Localized: Cancer is confined to the primary site (30, 31).

Regional: Cancer has spread beyond the primary site to nearby lymph nodes or tissues (30, 31).

Distant: Cancer has spread to distant organs or remote lymph nodes (30, 31).

### Independent variable of interest

The primary variable of interest was the implementation of Medicaid expansion under the ACA. This variable was operationalized as a categorical variable representing two time periods: pre-ACA (2011-2015) and post-ACA (2017-2021). Additionally, the interaction between the Medicaid implementation period and the state expansion status (Georgia vs. Louisiana) was examined to assess the differential impact of the policy on breast cancer outcomes across states.

### Covariates

Covariates included demographic factors such as age at diagnosis (categorized as 18-44 years, 45-65 years, and >65 years), race/ethnicity (classified as non-Hispanic White, non-Hispanic Black, Hispanic, non-Hispanic Asian/Pacific Islander), and marital status. Socioeconomic status was assessed using household median income, categorized as <$75,000 and ≥$75,000. Clinical factors such as disease stage at presentation (localized, regional and distant metastasis) and treatment modalities (surgery, chemotherapy, radiation) were also considered as covariates (32, 33).

### Theoretical model: Andersen behavioral model

This study uses the Andersen Behavioral Model of Health Services (34, 35) Use to explore the impact of the ACA and Medicaid expansion on breast cancer mortality, comparing Louisiana (which implemented Medicaid expansion in 2016) and Georgia (which has not). The model categorizes factors into predisposing, enabling, and need factors:

Predisposing Factors: These include demographics such as age (18-45, 45-64 years), race/ethnicity (White, Black, Hispanic, etc.), and marital status (single, married, etc.), which influence healthcare-seeking behavior.

Enabling Factors: These are resources that facilitate access to care, including income (<70K, ≥70K), metropolitan status (rural, small, medium, large metropolitan), and the state of residence (Louisiana vs. Georgia), reflecting the impact of Medicaid expansion.

Need Factors: These include clinical variables such as cancer grade (localized, regional, distant) (36), and the receipt of treatments like surgery, chemotherapy, and radiotherapy, all of which influence breast cancer outcomes.

### Difference-in-differences specification

The present study used a Difference-in-Differences (DID) model to estimate the impact of Medicaid expansion on breast cancer outcomes, overall survival, overall deaths, and disease stage at presentation by comparing Louisiana (treatment group, which implemented Medicaid expansion) to Georgia (control group, which did not) during the pre-ACA and post-ACA periods. The variable ACA was set to 1 for the post-ACA period (2017–2021) and 0 for the pre-ACA period (2011–2015). The variable State was defined as 1 if the observation was from Louisiana (expansion state) and 0 if from Georgia (non-expansion state).

The DID model is specified as:

*y*=*X*β+β_1_·ACA+β_2_·State + β_12_· (ACA X State) +u (37).

*y* represents the breast cancer outcome of interest (e.g., survival, overall mortality, or disease stage at presentation).

*X* includes covariates such as age, race, marital status, and treatment modalities.

β_1_ captures the difference in outcomes between the pre- and post-ACA periods across both states.

β_2_ captures the baseline difference between Louisiana and Texas.

β_12_ represents the effect of Medicaid expansion on breast cancer outcomes.

The interaction term ACA × State (i.e., β_12_) estimates the difference in the change in outcomes between Louisiana (the expansion state) and Georgia (the non-expansion state) from the pre- to the post-ACA period. This term provides the key estimate of the impact of Medicaid expansion (37).

### Sub-analysis: impact of chemotherapy on CSS and OS

The procedure was conducted using Inverse Probability Weighted Regression Adjustment (IPWRA) to evaluate the impact of chemotherapy on overall survival (OS) and cancer-specific mortality. We implemented the teffects ipwra command in Stata, which combines the benefits of both regression adjustment and propensity score weighting to estimate the Average Treatment Effect (ATE) of chemotherapy on survival outcomes. The treatment model was specified using a logit regression, with chemotherapy as the treatment variable. Covariates included the status of Medicaid expansion (EXPAND), post-ACA period (ACA), age (AGE2), race (i.Race), marital status (i.Married), income (Income2), metropolitan status (i.Metro), hormone receptor status (HER2, PRS, Estrogen), tumor characteristics (primary, Lateral), and cancer grade (i.Grade). This approach ensures that potential confounders are controlled, providing robust estimates of the effect of chemotherapy on survival outcomes in a population-based cohort.

### Statistical analysis

Descriptive statistics were used to summarize the characteristics of the study population. Univariate analysis, employing chi-square tests for categorical variables, was conducted to examine the distribution of study variables across different groups. Statistical analyses were performed using appropriate regression models, such as multinomial logistic regression for the disease stage and Cox proportional hazards regression for time-to-event outcomes for the CSS and OS. A margins plot was utilized to visualize the adjusted probabilities of each outcome across states and time periods. Statistical significance was determined using two-tailed tests with a predetermined alpha level (e.g., p < 0.05). All analyses were conducted using STATA 16 statistical software package.

#### Baseline Characteristics

**Table 1:** Baseline characteristics of women with breast cancer in Georgia and Louisiana Pre-ACA and Post-ACA (2011-2021).

Variables	Pre-ACA (n=32,220)			Post-ACA (n=30,161)		
	Georgia (n= 22,644)	Louisiana (n= 9,576)	P-value	Georgia (n= 20,635)	Louisiana (n=9,576)	p-value
**Age, year, Mean ± SD**	52.3 ± 8.7	52.6 ± 8.9	0.003	52.0 ± 8.6	52.7 ± 8.5	<0.001
Age(years)			0.87			< 0.001
18-45yrs.	4,345 (19.2%)	1,845 (19.3%)		4,107 (19.9%)	1,660 (17.4%)	
45-64yrs.	18,299 (80.8%)	7,731 (80.7%)		16,528 (80.1%)	7,866 (82.6%)	
**Race/Ethnicity**			<0.001			<0.001
White	12,559 (55.5%)	5,806 (60.6%)		12,217 (59.2%)	5,923 (62.2%)	
Black	7,781 (34.4%)	3,308 (34.5%)		6,876 (33.3%)	3,268 (34.3%)	
Hispanic	1,344 (5.9%)	275 (2.9%)		934 (4.5%)	204 (2.1%)	
Asian or Pacific Islanders	846 (3.7%)	141 (1.5%)		559 (2.7%)	103 (1.1%)	
Naive American	41 (0.2%)	27 (0.3%)		23 (0.1%)	16 (0.2%)	
Unknown	73 (0.3%)	19 (0.2%)		26 (0.1%)	12 (0.1%)	
**Marital Status**			<0.001			<0.001
Single	4,557 (20.2%)	2,375 (24.9%)		3,714 (18.0%)	2,097 (22.0%)	
Married	13,199 (58.6%)	5,172 (54.2%)		11,941 (58.0%)	5,191 (54.5%)	
Widowed	802 (3.6%)	416 (4.4%)		859 (4.2%)	437 (4.6%)	
Divorced	2,669 (11.9%)	1,128 (11.8%)		2,712 (13.2%)	1,098 (11.5%)	
Separated	356 (1.6%)	135 (1.4%)		319 (1.6%)	132 (1.4%)	
Unknown	940 (4.2%)	326 (3.4%)		1,056 (5.1%)	562 (5.9%)	
**Metropolitan Status**			<0.001			< 0.001
Rural areas not adjacent to Metro	866 (4.3%)	192 (3.3%)		855 (4.1%)	250 (2.6%)	
Rural areas adjacent to Metro	2,602 (12.9%)	1,159 (19.7%)		2,472 (12.0%)	1,176 (12.4%)	
Small Metropolitan	3,235 (16.1%)	1,700 (28.9%)		2,831 (13.7%)	1,640 (17.2%)	
Medium Metropolitan	13,422 (66.7%)	2,841 (48.2%)		2,362 (11.5%)	3,618 (38.0%)	
Large Metropolitan				12,115 (58.7%)	2,842 (29.8%)	
**Household Median Income**			< 0.001			< 0.001
<70K	8,962 (39.6%)	8,216 (85.8%)		11,877 (57.6%)	8,328 (87.4%)	
≥70K	13,682 (60.4%)	1,360 (14.2%)		8,758 (42.4%)	1,198 (12.6%)	
**Surgery**			< 0.001			< 0.001
Yes, n (%)	20,361 (90.0%)	8,690 (91.3%)		18,861 (91.5%)	8,698 (93.5%)	
None/Unknown	2,262 (10.0%)	830 (8.7%)		1,745 (8.5%)	608 (6.5%)	
**Chemotherapy**			0.8			0.961
Yes, n (%)	12,194 (53.9%)	5,142 (53.7%)		11,957 (58.0%)	5,517 (57.9%)	
None/Unknown	10,450 (46.2%)	4,434 (46.3%)		8,678 (42.1%)	4,009 (42.1%)	
**Radiotherapy**			0.003			<0.001
Yes, n (%)	12,638 (59.0%)	5,294 (57.2%)		10,968 (57.2%)	4,988 (54.1%)	
None/Unknown	8,799 (41.1%)	3,970 (42.9%)		8,208 (42.8%)	4,225 (45.9%)	
**Disease Stage at Presentation**			< 0.001			< 0.001
Localized	13,482 (59.5%)	5,830 (60.9%)		12,107 (58.7%)	5,644 (59.3%)	
Regional	7,326 (32.4%)	3,025 (31.6%)		6,889 (33.4%)	3,135 (32.9%)	
Distant	1,627 (7.2%)	674 (7.0%)		1,375 (6.7%)	686 (7.2%)	
Unknown	209 (0.9%)	47 (0.5%)		264 (1.3%)	61 (0.6%)	
Survival Outcomes						
Cancer-Specific Mortality (CSS)	1,256 (5.6%)	597 (6.2%)	0.016	3,163 (15.4%)	1,545 (16.3%)	0.041
Overall Deaths (OS)	1,660 (7.3%)	799 (8.34%)	0.002	4,331 (21.0%)	2,158 (22.7%)	0.001
4-Year Survival (CSS)	91.20%	90.40%	0.77	90.10%	89.60%	0.56
4-Year Survival (OS)	88.40%	87.40%	0.56	87.70%	86.70%	0.99

The baseline characteristics of women with breast cancer in Georgia and Louisiana during the pre-ACA (2011–2015) and post-ACA (2017–2021) periods highlight significant demographic and clinical differences between the states (**Table 1**). The study population consisted of 62,381 women, with 32,220 in the pre-ACA period and 30,161 in the post-ACA period. The mean age was slightly higher in Louisiana compared to Georgia in both periods (pre-ACA: 52.6 ± 8.9 vs. 52.3 ± 8.7, p=0.003; post-ACA: 52.7 ± 8.5 vs. 52.0 ± 8.6, p<0.001). The majority of women were aged 45-64 years in both states and time periods. Racial and ethnic distributions varied significantly, with a higher proportion of White women in Louisiana (pre-ACA: 60.6% vs. 55.5% in Georgia; post-ACA: 62.2% vs. 59.2% in Georgia, p<0.001), while Georgia had a larger proportion of Hispanic and Asian/Pacific Islander women in both periods.

#### Socioeconomic and Metropolitan Status

In Louisiana, a significantly higher proportion of women reported a household income of less than $70K compared to Georgia (pre-ACA: 85.8% vs. 39.6%, p<0.001; post-ACA: 87.4% vs. 57.6%, p<0.001) (**Table 1**). Georgia, on the other hand, had a higher percentage of women in the ≥$70K income category (pre-ACA: 60.4% vs. 14.2% in Louisiana; post-ACA: 42.4% vs. 12.6% in Louisiana, p<0.001). Metropolitan status also varied significantly, with the majority of women in Georgia residing in large metropolitan areas in the post-ACA period (58.7% vs. 29.8% in Louisiana, p<0.001), while Louisiana had a higher proportion of women in small and rural metropolitan areas.

#### Treatment Modalities and Disease Stage at Presentation

The treatment patterns showed significant variations between states (**Table 1**). Surgical rates were slightly higher in Louisiana during both periods (pre-ACA: 91.3% vs. 90.0% in Georgia; post-ACA: 93.5% vs. 91.5% in Georgia, p<0.001). Chemotherapy usage remained comparable between the states across both periods, while radiotherapy use was higher in Georgia (pre-ACA: 59.0% vs. 57.2% in Louisiana, p=0.003; post-ACA: 57.2% vs. 54.1%, p<0.001). Regarding disease stage at presentation, the proportion of women diagnosed at a localized stage was similar between states and periods (pre-ACA: 59.5% in Georgia vs. 60.9% in Louisiana; post-ACA: 58.7% in Georgia vs. 59.3% in Louisiana, p<0.001). However, there was a slight increase in distant-stage disease in Louisiana post-ACA (7.2% vs. 6.7% in Georgia, p<0.001).

#### Factors Associated with Cancer-Specific Survival

**Table 2:** Factors associated with cancer specific survival among women with breast cancers.

	Haz. Ratio	Std. Err.	z	P>z	95% CI	
Medicaid Expansion						
Louisiana (ref. Georgia)	1.070	0.070	1.040	0.297	0.942	1.216
Post-ACA (ref. Pre-ACA)	1.216	0.049	4.900	0.000	1.125	1.315
State X ACA						
Louisiana X Post-ACA	0.953	0.069	-0.670	0.502	0.827	1.098
Age(years)						
18-45yrs.	Reference					
45-64yrs.	1.047	0.035	1.370	0.172	0.980	1.117
Race/Ethnicity						
White	Reference					
Black	1.474	0.043	13.230	0.000	1.392	1.561
Hispanic	1.054	0.076	0.730	0.463	0.915	1.215
Asian/Pacific Islander	0.893	0.093	-1.090	0.276	0.729	1.094
Native American	1.202	0.492	0.450	0.653	0.539	2.679
Unknown	0.553	0.248	-1.320	0.187	0.230	1.332
Household Median Income						
<70K	Reference					
≥70K	0.962	0.036	-1.060	0.291	0.894	1.034
Metropolitan Status						
Rural areas not adjacent to Metro	Reference					
areas adjacent to metropolitans	0.861	0.061	-2.140	0.033	0.750	0.988
Small Metropolitan	0.900	0.062	-1.530	0.125	0.787	1.030
Medium Metropolitan	0.792	0.057	-3.240	0.001	0.688	0.912
Large Metropolitan	0.747	0.050	-4.340	0.000	0.655	0.852
Marital Status						
Single	Reference					
Married	0.807	0.028	-6.300	0.000	0.754	0.862
Widowed	1.075	0.069	1.140	0.255	0.949	1.219
Divorced	0.923	0.042	-1.750	0.081	0.844	1.010
Separated	0.960	0.093	-0.420	0.674	0.794	1.161
Unknown	0.869	0.056	-2.200	0.028	0.767	0.985
Disease Stage at Presentation						
Localized	Reference					
Regional	3.311	0.126	31.340	0.000	3.072	3.568
Distant	12.315	0.611	50.570	0.000	11.173	13.574
Missing	3.233	0.336	11.290	0.000	2.637	3.963
Treatment Modalities						
Surgery	0.325	0.014	-26.290	0.000	0.299	0.354
Chemotherapy	1.341	0.046	8.610	0.000	1.255	1.434
Radiation	0.834	0.025	-6.190	0.000	0.787	0.883

The Cox regression analysis indicated that the implementation of Medicaid expansioning Louisiana did not significantly affect CSS compared to Georgia (HR=1.070, 95% CI: 0.942-1.216, p=0.297) (**Table 2**). However, survival worsened in the post-ACA period (HR=1.216, 95% CI: 1.125-1.315, p<0.001), regardless of state. The interaction term between state and the post-ACA period was not significant (HR=0.953, 95% CI: 0.827-1.098, p=0.502), suggesting no differential effect of Medicaid expansion on survival between Louisiana and Georgia.

#### Demographic and Socioeconomic Factors

Age was not a significant predictor of cancer-specific mortality, but race/ethnicity had a substantial impact (**Table 2**). Black women had a significantly higher hazard of death compared to White women (HR=1.474, 95% CI: 1.392-1.561, p<0.001). Other racial/ethnic groups, such as Hispanics and Native Americans, did not show a significant difference from the reference group. Women in higher-income households (≥$70K) showed no significant survival benefit (HR=0.962, 95% CI: 0.894-1.034, p=0.291), but married women had a lower hazard of death (HR=0.807, 95% CI: 0.754-0.862, p<0.001) compared to single women.

#### Disease Stage, Metropolitan Status, and Treatment Modalities

Advanced disease stage was the strongest predictor of cancer-specific mortality, with the highest hazard observed in women with distant-stage disease (HR=12.315, 95% CI: 11.173-13.574, p<0.001) (**Table 2**). Women residing in large metropolitan areas had a significantly lower hazard of death (HR=0.747, 95% CI: 0.655-0.852, p<0.001) compared to those in rural areas. Treatment modalities also influenced survival, with surgery (HR=0.325, 95% CI: 0.299-0.354, p<0.001) and radiation (HR=0.834, 95% CI: 0.787-0.883, p<0.001) associated with improved outcomes, whereas chemotherapy was linked to an increased hazard of death (HR=1.341, 95% CI: 1.255-1.434, p<0.001).

#### Factors Associated with Overall Deaths

**Table 3:** Factors associated with overall deaths among women with breast cancers.

	Haz. Ratio	Std. Err.	z	P>z	95% CI	
Medicaid Expansion						
Louisiana (ref. Georgia)	1.036	0.058	0.630	0.529	0.928	1.157
Post-ACA (ref. Pre-ACA)	1.113	0.039	3.070	0.002	1.039	1.192
State X ACA						
Louisiana X Post-ACA	0.994	0.062	-0.100	0.920	0.880	1.123
Age(years)						
18-45yrs.	Reference					
45-64yrs.	1.218	0.037	6.540	0.000	1.148	1.293
Race/Ethnicity						
White	Reference					
Black	1.406	0.035	13.580	0.000	1.338	1.477
Hispanic	1.055	0.066	0.840	0.399	0.932	1.193
Asian/Pacific Islander	0.819	0.077	-2.130	0.034	0.681	0.985
Native American	1.521	0.460	1.390	0.165	0.841	2.750
Unknown	0.526	0.199	-1.700	0.090	0.250	1.106
Household Median Income						
<70K	Reference					
≥70K	0.909	0.029	-2.990	0.003	0.854	0.968
Metropolitan Status						
Rural areas not adjacent to Metro						
Rural areas adjacent to Metro	0.893	0.053	-1.900	0.057	0.795	1.003
Small Metropolitan	0.908	0.053	-1.670	0.095	0.810	1.017
Medium Metropolitan	0.789	0.048	-3.900	0.000	0.700	0.889
Large Metropolitan	0.738	0.042	-5.330	0.000	0.660	0.825
Marital Status						
Single	Reference					
Married	0.735	0.022	-10.500	0.000	0.694	0.778
Widowed	1.143	0.060	2.560	0.010	1.032	1.266
Divorced	0.917	0.035	-2.250	0.025	0.851	0.989
Separated	0.977	0.081	-0.280	0.781	0.831	1.149
Unknown	0.840	0.046	-3.220	0.001	0.755	0.934
Disease Stage at Presentation						
Localized						
Regional	2.439	0.073	29.830	0.000	2.300	2.586
Distant	7.398	0.312	47.530	0.000	6.812	8.034
Missing	2.145	0.190	8.600	0.000	1.803	2.553
Treatment Modalities						
Surgery	0.318	0.012	-30.320	0.000	0.296	0.343
Chemotherapy	1.143	0.031	4.850	0.000	1.083	1.206
Radiation	0.812	0.020	-8.350	0.000	0.773	0.853

The Cox regression analysis for overall deaths indicated that the implementation of Medicaid expansion in Louisiana did not significantly impact overall survival compared to Georgia (HR=1.036, 95% CI: 0.928-1.157, p=0.529) (**Table 3**). However, the hazard of death was significantly higher in the post-ACA period compared to the pre-ACA period (HR=1.113, 95% CI: 1.039-1.192, p=0.002). The interaction term between state and post-ACA period was not significant (HR=0.994, 95% CI: 0.880-1.123, p=0.920), suggesting that the effect of the ACA on overall survival did not differ significantly between Louisiana and Georgia.

#### Demographic and Socioeconomic Factors

Age and race/ethnicity were significant predictors of overall survival (**Table 3**). Women aged 45-64 years had a higher hazard of death compared to the reference group (18-45 years) (HR=1.218, 95% CI: 1.148-1.293, p<0.001). Black women had a significantly increased hazard of death compared to White women (HR=1.406, 95% CI: 1.338-1.477, p<0.001). Household income was also associated with survival, as women from higher-income households (≥$70K) had a lower hazard of death (HR=0.909, 95% CI: 0.854-0.968, p=0.003) compared to those with an income of less than $70K. Metropolitan status showed a protective effect, with women residing in large metropolitan areas having a significantly lower hazard of death (HR=0.738, 95% CI: 0.660-0.825, p<0.001) compared to those in rural areas.

#### Disease Stage, Marital Status, and Treatment Modalities

Advanced disease stage was a strong predictor of overall mortality, with women presenting with distant-stage disease having a significantly higher hazard of death (HR=7.398, 95% CI: 6.812-8.034, p<0.001) compared to those with localized disease (**Table 3**). Marital status also influenced survival, with married women exhibiting a lower hazard of death (HR=0.735, 95% CI: 0.694-0.778, p<0.001) compared to single women, while widowed women had an elevated hazard of death (HR=1.143, 95% CI: 1.032-1.266, p=0.010). Among treatment modalities, surgery was associated with a significant reduction in mortality risk (HR=0.318, 95% CI: 0.296-0.343, p<0.001), while chemotherapy was associated with an increased hazard of death (HR=1.143, 95% CI: 1.083-1.206, p<0.001). Radiation therapy significantly improved overall survival (HR=0.812, 95% CI: 0.773-0.853, p<0.001).

#### Comparison of Treatment Patterns Between Georgia and Louisiana

**Table 4:** Change in treatment patterns of women with breast cancers.

Variables	Pre-ACA (n=32,220)			Post-ACA (n=30,161)		
	Georgia	Louisiana	*p*	Georgia	Louisiana	*p*
	(n=22,644)	(n=9,576)		(n=20,635)	(n=9,526)	
**Surg/Rad Sequence**			< 0.001			<0.001
Intraoperative rad with other rad before surgery	30 (0.13%)	3 (0.03%)		38 (0.18%)	2 (0.02%)	
Intraoperative radiation	113 (0.50%)	0 (%)		84 (0.41%)	4 (0.04%)	
No radiation and/or no surgery; unknown	10,157 (44.86%)	4,390 (45.84%)		9,329 (45.21%)	4,530 (47.55%)	
Radiation after surgery	12,224 (53.98%)	5,099 (53.25%)		11,061 (53.60%)	4,891 (51.34%)	
Radiation before and after surgery	35 (0.15%)	39 (0.41%)		25 (0.12%)	33 (0.35%)	
Radiation prior to surgery	51 (0.23%)	41 (0.43%)		75 (0.36%)	44 (0.46%)	
Sequence unknown but both were given	24 (0.11%)	2 (0.02%)		16 (0.08%)	22 (0.23%)	
Surgery both before and after radiation	10 (0.04%)	2 (0.02%)		7 (0.03%)	0 (0.00%)	
**Systemic/Sur Seq**			<0.001			<0.001
Intraop systemic rx & oth systemic rx b	5 (0.02%)	4 (0.04%)		5 (0.02%)	1 (0.01%)	
Intraoperative systemic therapy	2 (0.01%)	1 (0.01%)		11 (0.05%)	2 (0.02%)	
No systemic therapy and/or surgical pro	3,897 (17.21%)	1,671 (17.45%)		3,639 (17.64%)	2,026 (21.27%)	
Sequence unknown	4 (0.02%)	10 (0.10%)		9 (0.04%)	33 (0.35%)	
Surgery both before and after systemic	1,004 (4.43%)	229 (2.39%)		468 (2.27%)	84 (0.88%)	
Systemic therapy after surgery	12,849 (56.74%)	5,632 (58.81%)		12,964 (62.83%)	6,018 (63.17%)	
Systemic therapy before surgery	2,425 (10.71%)	777 (8.11%)		1,928 (9.34%)	699 (7.34%)	
Systemic therapy both before and after	2,458 (10.85%)	1,252 (13.07%)		1,611 (7.81%)	663 (6.96%)	

Treatment patterns differed slightly between Georgia and Louisiana in both the pre- and post-ACA periods (**Table 4**). In the pre-ACA period, the majority of patients in both states received radiation after surgery (Georgia: 53.98% vs. Louisiana: 53.25%), while a significant proportion had no radiation or surgery (Georgia: 44.86% vs. Louisiana: 45.84%). Similar trends persisted in the post-ACA period, with slightly increased proportions of patients receiving no radiation or surgery in both states (Georgia: 45.21% vs. Louisiana: 47.55%, p<0.001). Interestingly, the use of intraoperative radiation was more frequent in Georgia than in Louisiana across both periods, though the overall numbers remained low.

For systemic therapy, the majority of women in both states received systemic therapy after surgery in the pre-ACA period (Georgia: 56.74% vs. Louisiana: 58.81%, p<0.001). However, this trend increased significantly in the post-ACA period, with both states showing higher utilization of systemic therapy after surgery (Georgia: 62.83% vs. Louisiana: 63.17%). The proportion of women receiving systemic therapy both before and after surgery decreased post-ACA in both states (Georgia: 7.81% vs. 6.96% in Louisiana) compared to the pre-ACA period (Georgia: 10.85% vs. 13.07% in Louisiana). Additionally, the number of women receiving systemic therapy before surgery declined post-ACA (Georgia: 9.34% vs. Louisiana: 7.34%, p<0.001).

Overall, the shift in treatment patterns post-ACA, particularly the increased reliance on systemic therapy after surgery, highlights evolving treatment strategies in both states, although the changes were more pronounced in Louisiana.

#### Baseline Characteristics of Breast Cancer Patients Receiving Chemotherapy Before and After Propensity Score Matching

**Table 5:** Baseline characteristics of breast cancer patients receiving chemotherapy before and after propensity score matching.

Covariates	Before matching			After matching		
	Chemo (%)	Not Chemo (%)	% Bias	Chemo (%)	Not Chemo (%)	% Bias
Louisiana	30.6	30.6	0.1	27.3	27.2	0.2
Post-ACA	50.2	46.0	8.4	55.0	55.9	-1.8
Age (45-64yrs.)	75.2	87.9	-33.2	75.3	79.6	-11.3
Race/Ethnicity						
Non-Hispanic White	53.3	65.2	-24.6	53.3	51.7	3.3
Non-Hispanic Black	39.1	27.7	24.4	38.7	42.0	-7.1
Hispanic	4.8	3.9	4.5	5.0	3.8	5.8
Asian/Pacific Islander	2.6	2.7	-0.8	2.7	2.3	2.4
Native Americans	0.2	0.2	0.3	0.2	0.1	2.7
Marital Status						
Single	22.7	17.6	12.5	22.4	23.7	-3.4
Married	56.0	58.6	-5.4	56.4	55.0	2.8
Widowed	3.8	4.5	-3.3	3.8	3.4	1.6
Divorced	12.1	12.1	-0.0	12.1	11.9	0.5
Separated	1.7	1.3	3.2	1.7	1.2	4.1
Income >70K	38.1	41.7	-7.5	40.6	40	1.3
Metropolitan Status						
Rural Areas	13.4	12.6	2.5	13.6	13.7	-0.3
Small Metropolitan	16.9	16.2	1.8	16.9	16.3	1.5
Medium Metropolitan	11.9	11	2.8	11.1	10.6	1.5
Large Metropolitan	53.9	56.5	-5.3	54.6	55.1	-1.0
Hormone Receptor Status						
HER2+	27.3	0.6	57.4	27.1	28.7	-4.6
PRS+	53.6	85.2	-73.1	53.6	51.2	5.5
Estrogen+	66.6	92.9	-69.2	66.8	65.6	3.1
Primary	89.9	87.1	8.7	90.1	89.1	3.2
Lateral	49.1	49.3	-0.3	49.1	49.7	-1.2
Stage						
Localized	43.1	80.0	-82.0	43.4	44.6	-2.5
Regional	47.3	14.2	76.8	47.7	45.1	5.9
Distant metastasis	9.2	4.1	20.7	8.7	10.1	-5.6

Before propensity score matching, significant imbalances were observed across several covariates between patients who received chemotherapy and those who did not (**Table 5**). For instance, patients in the chemotherapy group had a higher proportion of Non-Hispanic Black individuals (39.1% vs. 27.7%, % bias = 24.4) and were less likely to have localized-stage cancer (43.1% vs. 80%, % bias = -82). Other notable disparities included hormone receptor status such as HER2 (27.3% vs. 6.6%, % bias = 57.4), PRS (53.6% vs. 85.2%, % bias = -73.1), and Estrogen receptor positivity (66.6% vs. 92.9%, % bias = -69.2). After matching, these imbalances were substantially reduced, achieving a closer balance between the two groups. For example, the % bias for the proportion of Non-Hispanic Black individuals dropped to -7.1%, and for localized-stage cancer, the % bias reduced to -2.5%. These results indicate that propensity score matching effectively minimized baseline differences, allowing for a more accurate comparison of outcomes between patients who received chemotherapy and those who did not.

#### Effect of Chemotherapy on Cancer-Specific Mortality and Overall Survival

**Table 6:** Effect of chemotherapy on cancer-specific mortality and overall survival.

Outcome	Treatment Group	Coeff. (ATE)	Std. Err	z-value	p-value	95% CI
Cancer-specific mortality rate	Chemotherapy vs. No Chemotherapy	-0.084	0.011	-7.43	<0.001	-0.106, -0.062
Cancer-specific mortality rate	No Chemotherapy	0.199	0.011	17.79	<0.001	0.177, 0.221
Overall mortality rate	Chemotherapy vs. No Chemotherapy	-0.105	0.012	-9.16	<0.001	-0.128, -0.083
Overall mortality rate	No Chemotherapy	0.256	0.011	22.47	<0.001	0.233, 0.278

This table shows the Inverse Probability Weighting (IPW) estimates of chemotherapy’s impact on cancer-specific and overall mortality. Chemotherapy significantly reduced cancer-specific mortality (ATE: -0.084, 95% CI: -0.106, -0.062, p < 0.001) and overall mortality (ATE: -0.105, 95% CI: -0.128, -0.083, p < 0.001) compared to no chemotherapy.

The effect of chemotherapy on cancer-specific mortality and overall survival was evaluated using the Average Treatment Effect (ATE) (**Table 6**). Chemotherapy was significantly associated with a reduction in cancer-specific mortality, showing an 8.38 percentage point decrease compared to those who did not receive chemotherapy (ATE = -0.0838, SE = 0.0113, p < 0.001, 95% CI: -0.1059 to -0.0617). Similarly, chemotherapy was associated with a significant reduction in the risk of overall deaths by 10.53 percentage points (ATE = -0.1053, SE = 0.0115, p < 0.001, 95% CI: -0.1278 to -0.0828). The potential outcome mean (PO Mean) for cancer-specific mortality in the non-chemotherapy group was 19.92% (SE = 0.0112, 95% CI: 17.73% to 22.12%), while for overall deaths, the predicted risk was 25.55% (SE = 0.0114, 95% CI: 23.32% to 27.78%). These findings suggest that chemotherapy not only significantly reduces cancer-specific mortality but also lowers the risk of all-cause mortality in this population.

#### Impact of Medicaid Expansion on Cancer-Specific Deaths

**Table 7:** Cancer-specific mortality: DID Estimate.

	Coef.	Std. Err.	z	P>z	95% Confidence Interval	
All	-5.97	0.103	-0.580	0.561	-26.09	14.15
White	-4.97	0.085	-0.580	0.561	-21.73	11.78
Black	-7.33	0.126	-0.580	0.561	-32.04	17.38
Hispanic	-5.24	0.090	-0.580	0.561	-22.92	12.43
Asian or Pacific Islander	-4.44	0.076	-0.580	0.561	-19.43	10.55
Naive American	-5.98	0.106	-0.560	0.572	-26.72	14.77

This table shows the estimated coefficients (Coef.), standard errors (Std. Err.), z-scores (z), p-values (P>z), and 95% confidence intervals ([95% Conf. Interval]) from the difference-in-differences analysis evaluating the impact of Medicaid expansion on cancer-specific mortality among women with breast cancer, stratified by race/ethnicity (All, White, Black, Hispanic, Asian or Pacific Islander, and Native American). Coefficients represent the differential change in cancer-specific mortality in Louisiana compared to Georgia from the pre- to post-ACA periods.

The DID analysis did not show a significant change in **CSS** between Louisiana and Georgia across the pre- and post-ACA periods (**Table 7**). The overall coefficient for the difference in changes between the two states was -5.97 (95% CI: -26.09 to 14.15, p = 0.561), indicating no significant improvement or worsening of survival rates in Louisiana compared to Georgia. When stratified by race, similar patterns were observed across racial and ethnic groups, with coefficients ranging from -4.44 for Asian or Pacific Islanders (95% CI: -19.43 to 10.55, p = 0.561) to -7.33 for Black women (95% CI: -32.04 to 17.38, p = 0.561). The lack of significant change across all racial subgroups suggests that Medicaid expansion in Louisiana did not differentially impact CSS compared to Georgia, regardless of race.

#### Impact of Medicaid Expansion on Overall Mortality

**Table 8:** Overall deaths: DID Estimates.

	Coef.	Std. Err.	z	P>z	95% Confidence Interval	
All	-0.26	0.05436	-0.05	0.962	-10.91	10.39
White	-0.22	0.046551	-0.05	0.962	-9.35	8.9
Black	-0.31	0.065441	-0.05	0.962	-13.14	12.51
Hispanic	-0.23	0.04909	-0.05	0.962	-9.86	9.39
Asian or Pacific Islander	-0.18	0.038117	-0.05	0.962	-7.65	7.29
Naive American	-0.34	0.070826	-0.05	0.962	-14.22	13.54

This table displays the estimated coefficients (Coef.), standard errors (Std. Err.), z-scores (z), p-values (P>z), and 95% confidence intervals ([95% Conf. Interval]) from the difference-in-differences analysis examining the impact of Medicaid expansion on overall mortality rates among women with breast cancer, stratified by race/ethnicity (All, White, Black, Hispanic, Asian or Pacific Islander, and Native American). Coefficients reflect the differential change in overall mortality in Louisiana compared to Georgia in the pre- and post-ACA periods.

The DID analysis revealed no significant changes in overall mortality between Louisiana and Georgia in the pre- and post-ACA periods (**Table 8**). The overall coefficient for the difference in changes between the two states was -0.26 (95% CI: -10.91 to 10.39, p = 0.962), indicating that Medicaid expansion did not result in a measurable difference in overall mortality trends between the two states. When examined by race, similar non-significant results were observed, with coefficients ranging from -0.18 for Asian or Pacific Islanders (95% CI: -7.65 to 7.29, p = 0.962) to -0.34 for Native Americans (95% CI: -14.22 to 13.54, p = 0.962). These findings suggest that the implementation of Medicaid expansion in Louisiana did not significantly influence overall mortality rates compared to Georgia, across all racial and ethnic groups.

#### Impact of Medicaid Expansion on Disease Stage at Presentation

**Table 9:** Disease stage at presentation: DID Estimates.

	Coef.	Std. Err.	z	P>z	[95% Conf.	Interval
Localized	-1.27	0.010	-1.222	0.222	-3.31	0.77
Regional	0.47	0.010	0.474	0.635	-1.48	2.43
Distant Metastasis	1.08	0.006	1.943	0.052	-0.01	2.17

The table presents the estimated coefficients (Coef.), standard errors (Std. Err.), z-scores (z), p-values (P>z), and 95% confidence intervals ([95% Conf. Interval]) for the difference-in-differences analysis evaluating changes in breast cancer stage at presentation (Localized, Regional, and Distant Metastasis). Coefficients represent the differential impact of Medicaid expansion on the likelihood of being diagnosed at each disease stage in Louisiana compared to Georgia.

The DID analysis showed no significant change in the proportion of patients presenting at localized or regional stages in Louisiana compared to Georgia following Medicaid expansion (**Table 9**). The coefficient for localized stage was -1.27 (95% CI: -3.31 to 0.77, p = 0.221), and for regional stage, the coefficient was 0.47 (95% CI: -1.48 to 2.43, p = 0.635), indicating no significant shifts in early-stage presentations. However, there was a marginally significant increase in the likelihood of distant metastasis at presentation (coefficient: 1.08, 95% CI: -0.01 to 2.17, p = 0.052), suggesting a possible trend toward more advanced stage diagnoses post-ACA in Louisiana relative to Georgia, though this finding did not reach conventional levels of statistical significance.

## Discussion

In this study, we sought to evaluate the causal impact of Medicaid expansion in Louisiana, which implemented the policy in 2016, by comparing cancer-specific survival (CSS) and overall mortality among women with breast cancer aged 18-64 years to those in Georgia, a state that has not yet implemented Medicaid expansion. This age group was chosen because individuals aged 18-64 (38) are more likely to benefit from Medicaid expansion, given that individuals over 65 are primarily covered by Medicare (39). Our analysis, which was stratified by income (households earning below the median income of $70,000 in both states), focused on changes in CSS, OS, and disease stage at presentation.

## Main findings

We found no statistically significant differences in CSS between Louisiana and Georgia following Medicaid expansion. Specifically, individuals in Louisiana experienced an estimated 6% reduction in CSS relative to their counterparts in Georgia. However, the confidence intervals were wide, ranging from a 26% reduction to a 14.2% increase, indicating high variability and uncertainty. Similarly, no significant differences were observed across different race/ethnic groups, though there was a non-significant trend toward a 7.3% reduction in CSS for non-Hispanic Black women in Louisiana (95% CI: -32.0% to +17.4%).

A potential explanation for the lack of statistical significance is the latency of breast cancer (40, 41). Breast cancer is a disease with a relatively long progression and treatment course, and differences in survival outcomes might take longer to manifest (40, 42, 43). This delayed impact could obscure any immediate differences between the states within the study period.

Moreover, while there was no significant increase in OS, the trends in Louisiana were positive, showing possible improvements in both CCS and OS. This trend, although not statistically significant, suggests that Medicaid expansion may lead to improved outcomes over time (44, 45).

## Disease stage at presentation

We examined cancer staging at presentation, classifying breast cancer cases as localized, regional, or distant metastatic. Similar to CSS and OS, there were no significant differences in the stage at presentation between the two states following Medicaid expansion. This may be partly due to the pre-existing national programs such as the National Breast and Cervical Cancer Early Detection Program (46), which predates the ACA and may have blunted the impact of Medicaid expansion on cancer screening and early detection.

## Comparison with existing literature

Our findings align with some existing literature that also reports no immediate, significant effect of Medicaid expansion on cancer-specific mortality in the short term (47). For instance, previous studies examining cancer outcomes post-Medicaid expansion have found mixed results, with some studies indicating improvements in early detection and care access but no statistically significant change in survival outcomes during the first few years post-expansion (48, 49).

One key explanation in the literature is that the benefits of policy changes like Medicaid expansion may take time to manifest, particularly for diseases with longer survival curves, such as cancer (10, 48, 50). For instance, research has shown that while expansion improves access to care, it may take several years for this to translate into improved long-term survival, as newly insured patients may not access care immediately, and cancer treatment is often a lengthy process (50, 51). Moreover, socioeconomic factors and pre-existing disparities may further complicate short-term outcomes (52, 53).

The absence of significant differences in survival outcomes between Louisiana and Georgia post-Medicaid expansion could be attributed to several interconnected factors. First, the latent nature of breast cancer might delay observable impacts on survival outcomes. Breast cancer is a disease with a prolonged progression, and changes in mortality outcomes may not manifest until several years after diagnosis and treatment initiation (54). Research shows that it often takes time for the benefits of improved access to healthcare, such as that provided by Medicaid expansion, to influence long-term outcomes like cancer survival (10). Thus, the full effects of Medicaid expansion in Louisiana may not yet be apparent within the study period. Moreover, the capacity of Louisiana’s healthcare system may have played a role. Medicaid expansion often leads to an influx of newly insured individuals, and if the healthcare infrastructure in Louisiana was already strained, particularly in rural or underserved areas, this could limit access to timely and quality cancer care, delaying the potential benefits of the policy (10, 55).

Additionally, national programs predating the Affordable Care Act, such as the National Breast and Cervical Cancer Early Detection Program, may have already been effective in both states, reducing the observable impact of Medicaid expansion. Both Louisiana and Georgia likely benefited from these programs, which have been instrumental in providing low-income and uninsured women access to cancer screening and early detection services (44, 56). Furthermore, differences in baseline health conditions between the populations of Louisiana and Georgia could have influenced the results (57). If Louisiana had a higher burden of comorbidities such as obesity or hypertension, which are known to impact cancer outcomes, the Medicaid expansion may not have fully offset these pre-existing health disparities (58, 59). In addition, persistent racial and socioeconomic disparities likely played a significant role (60–62). Despite improved access to healthcare through Medicaid expansion, Black women continued to experience worse CSS compared to White women, highlighting that access alone does not address deeper social determinants of health, such as poverty, education, and systemic racism (63).

Lastly, limitations in the study design and data may have contributed to the lack of significant findings. The sample size, particularly for subgroups such as racial/ethnic minorities, may have been too small to detect meaningful differences, while variability in outcomes and wide confidence intervals could indicate that the analysis lacked sufficient statistical power (64). Furthermore, differences in how cancer data were recorded and classified between the two states may have introduced inconsistencies, masking real differences in outcomes (65). These methodological challenges, combined with the aforementioned factors, underscore the complexity of evaluating the impact of Medicaid expansion on cancer-specific outcomes (10, 48, 50). Longer-term studies with larger, more diverse samples are likely needed to fully capture the effects of Medicaid expansion on cancer survival.

## Chemotherapy and survival outcomes

In the initial Cox regression analysis, chemotherapy was paradoxically associated with worse cancer-specific and overall survival. This counterintuitive finding likely reflects confounding by indication, as patients receiving chemotherapy may have had more advanced disease or unfavorable prognostic factors, leading to poorer survival outcomes. Similar paradoxical associations due to confounding by indication have been observed in other observational studies of cancer treatments (66). However, after applying propensity score matching to balance these baseline differences, chemotherapy was associated with improved cancer-specific and overall survival. This suggests that when accounting for patient characteristics and treatment selection bias, chemotherapy indeed offers a survival benefit, consistent with its intended therapeutic effect (67–69). Our findings align with prior studies that have reported similar associations between chemotherapy and improved outcomes in appropriately matched cohorts, highlighting the importance of adjusting for baseline imbalances when assessing treatment efficacy (70–72). These results underscore the potential misinterpretation of chemotherapy effects without rigorous control for confounding and support the use of propensity score matching in observational analyses to yield more reliable estimates.

## Implications for policy

While the results of this study did not reach statistical significance, the trends suggest that Medicaid expansion may contribute to a gradual reduction in cancer-specific mortality and overall deaths. Policymakers should consider that the full effects of expansion might take longer to manifest in populations with complex, long-term health needs, such as cancer patients. Further, efforts to address social determinants of health, beyond just expanding access to healthcare are crucial for reducing racial and socioeconomic disparities in cancer outcomes.

## Study limitation

This study has several important limitations. First, although the SEER registry provides a robust source of cancer data, it is limited in capturing other clinical and socioeconomic variables that may impact cancer-specific outcomes, such as comorbidities, healthcare access, and treatment adherence. The SEER database also lacks detailed information on insurance status, limiting our ability to track changes in coverage following Medicaid expansion. Furthermore, SEER is observational in nature, and while our difference-in-differences approach helps mitigate some confounding factors, it does not establish definitive causality. Another key limitation is the follow-up period, which may be too short to observe the full long-term impact of Medicaid expansion on breast cancer outcomes, particularly given the long latency of the disease.

## Conclusion

The present study highlights the importance of a long-term perspective when evaluating the effects of Medicaid expansion on cancer survival. Continued monitoring and analysis are necessary to fully understand the potential benefits of Medicaid expansion for underserved populations, especially those facing socioeconomic barriers to care.

## Data Availability

The raw data supporting the conclusions of this article will be made available by the authors, without undue reservation.
